# Phase II trial of capecitabine plus modified cisplatin (mXP) as first-line therapy in Japanese patients with metastatic gastric cancer (KSCC1104)

**DOI:** 10.1007/s00280-016-3204-6

**Published:** 2016-12-10

**Authors:** Hironaga Satake, Masaaki Iwatsuki, Yoshikazu Uenosono, Takeshi Shiraishi, Hiroaki Tanioka, Hiroshi Saeki, Keishi Sugimachi, Dai Kitagawa, Mototsugu Shimokawa, Eiji Oki, Yasunori Emi, Yoshihiro Kakeji, Akihito Tsuji, Yoshito Akagi, Shoji Natsugoe, Hideo Baba, Yoshihiko Maehara, Kyushu Study Group of Clinical Cancer

**Affiliations:** 1 Department of Medical Oncology, Kobe City Medical Center General Hospital, Kobe, Japan; 2 Department of Gastroenterological Surgery, Graduate School of Medical Sciences, Kumamoto University, Kumamoto, Japan; 3 Department of Digestive Surgery, Breast and Thyroid Surgery, Kagoshima University Graduate School of Medical and Dental Sciences, Kagoshima, Japan; 4 Department of Medical Oncology, Matsuyama Red Cross Hospital, Matsuyama, Ehime Japan; 5 Department of Medical Oncology, Okayama Rosai Hospital, Okayama, Japan; 6 Department of Surgery and Science, Graduate School of Medical Sciences, Kyushu University, Fukuoka, Japan; 7 Department of Surgery, Kyushu University Beppu Hospital, Beppu, Japan; 8 Department of Gastrointestinal Surgery, Kyushu Central Hospital of the Mutual Aid Association of Public School Teachers, Fukuoka, Japan; 9 Clinical Research Institute, Cancer Biostatistics Laboratory, National Kyushu Cancer Center, Fukuoka, Japan; 10 Department of Surgery, Saiseikai Fukuoka General Hospital, Fukuoka, Japan; 11 Division of Gastrointestinal Surgery, Department of Surgery, Graduate School of Medicine, Kobe University, Kobe, Japan; 12 Department of Clinical Oncology, Faculty of Medicine, Kagawa University, Kagawa, Japan; 13 Department of Surgery, Kurume University School of Medicine, Kurume, Japan

**Keywords:** Gastric cancer, Modified XP regimen, Capecitabine, Cisplatin, Chemotherapy

## Abstract

**Purpose:**

Capecitabine plus cisplatin (XP) is a standard therapy for metastatic gastric cancer (mGC). However, while results from previous phase III trials suggested that the cisplatin dosage should be reduced in Japanese patients, no clinical data exist to support this. Here, we conducted a multicenter study to evaluate the efficacy and safety of modified XP (mXP) in Japanese patients with mGC.

**Methods:**

Patients with previously untreated mGC received mXP (cisplatin 60 mg/m^2^ on day 1 plus capecitabine 1000 mg/m^2^ twice daily on days 1–14) every 3 weeks. The primary endpoint was the Response Evaluation Criteria in Solid Tumors-confirmed overall response rate (ORR). A sample size of 40 was planned for a threshold ORR of 30% and an expected value of 50%, with a one-sided *α* of 0.05 and a beta of approximately 0.2.

**Results:**

Forty-two patients were enrolled. One patient did not fulfill the eligibility criteria; therefore, a total of 41 patients were assessed. The results were as follows: complete response in 2 patients, partial response in 16, stable disease in 14, progressive disease in 8, and no evaluation in 1. The confirmed ORR was 43.9% (95% confidence interval 28.7–59.1%). The median progression-free survival and median overall survival were 4.6 and 11.3 months, respectively. The most common grade 3 or 4 adverse events were neutropenia (37.5%), anemia (24.4%), anorexia (24.4%), and nausea (12.2%).

**Conclusions:**

First-line chemotherapy with mXP in Japanese patients with mGC did not reach its primary objective. However, it did show a promising response rate and an acceptable tolerability profile.

## Introduction

Gastric cancer is the third leading cause of cancer-associated deaths worldwide [1]. Once the disease becomes inoperable, the prognosis for gastric cancer is exceptionally poor. Most cases of inoperable advanced or metastatic gastric cancer (mGC) remain incurable, and median survival is only 11–14 months, even for patients who undergo chemotherapy [2–4].

The combination of fluoropyrimidine and cisplatin is used worldwide for the treatment of mGC [2, 3]. Capecitabine is an oral fluoropyrimidine that is activated in tumor tissue via a three-step enzymatic conversion that culminates in the generation of thymidine phosphorylase [5]. Capecitabine has been shown to be effective in the treatment of gastric cancer; it is also administered as a combination treatment with cisplatin [6, 7]. Capecitabine plus cisplatin (XP) is regarded as a standard therapy for mGC. Globally, doses in the XP regimen consist of capecitabine (1000 mg/m^2^ twice daily on days 1–14) plus cisplatin (80 mg/m^2^ on day 1) every 3 weeks [8]. For Japanese patients, however, there has been no phase I trial of the combination XP regimen in a 3-week cycle. The pivotal Avastin in Gastric Cancer (AVAGAST) trial, a global phase III study that focused on the benefit of adding bevacizumab to the XP regimen for advanced gastric cancer [9], included 94 Japanese patients who received XP alone. The median progression-free survival (PFS) in this Japanese XP group was equivalent to that of the overall chemotherapy group (5.7 vs. 5.3 months). In the AVAGAST trial, however, the cisplatin dose was reduced in the second cycle from 80 to 60 mg/m^2^ in about 50% of the Japanese patients. Furthermore, a cisplatin dose reduction due to adverse events was reported in 79.8% of Japanese patients during the treatment course [10]. Accordingly, a more feasible dose for Japanese patients is anticipated.

Here, we develop a modified XP (mXP) regimen to reduce toxicity while maintaining efficacy in the treatment of mGC. This study was designed to evaluate the efficacy and safety of mXP in the clinical care of Japanese patients with mGC, including elderly patients. To confirm the safety of mXP regimens in both academic and community oncology practices, we performed this study across multiple institutions in both academic and community practice settings.

## Materials and methods

### Eligibility criteria

Eligibility criteria were: age > 20 years; histologically confirmed human epidermal growth factor receptor type 2 negative unresectable or recurrent gastric cancer; Eastern Cooperative Oncology Group (ECOG) performance status <2; one or more measurable tumor lesions according to the Response Evaluation Criteria in Solid Tumors (RECIST) guidelines [11]; estimated life expectancy ≥3 months; and adequate organ function, as defined by hemoglobin (Hb) ≥9 g/dL, white blood cell count ≥3000/mm^3^ ≤ 12,000/mm^3^, absolute neutrophil count (ANC) ≥1500/mm^3^, platelet count ≥100,000/mm^3^, total bilirubin ≤2.0 mg/dL, serum transaminase level ≤100 U/L, serum creatinine level ≤1.50 mg/dL, and creatinine clearance ≥50 mL/min. Adjuvant chemotherapy was allowed if >6 months had elapsed between the end of the therapy and the registration. Exclusion criteria were as follows: contraindication to any drug contained in the chemotherapy regimen; evidence of prior history of platinum administration; insufficient oral intake; synchronous or previous malignancy other than carcinoma in situ; severe comorbidities; active bleeding from the digestive tract; uncontrolled infection; severe mental disorder; pregnancy or lactation; and brain metastasis.

This trial was carried out in accordance with the Helsinki Declaration and Good Clinical Practice guidelines and was approved by the institutional review boards of all participating institutions. All patients were required to give written informed consent before entering the study. The Kyushu Study Group of Clinical Cancer (KSCC) Data Center conducted the data management, central monitoring, and statistical analysis.

### Study design and treatment

Protocol treatment was defined as chemotherapy consisting of capecitabine and cisplatin. Patients received capecitabine (1000 mg/m^2^ twice daily on days 1–14) plus cisplatin (60 mg/m^2^ on day 1) every 3 weeks. Following capecitabine administration on days 1–14, there was a 1-week rest period. Treatment was repeated until disease progression, unacceptable toxicity, or withdrawal of consent.

To prevent chemotherapy-induced nausea and vomiting, the 5-hydroxytryptamine-3 receptor antagonist dexamethasone [9.9 mg, intravenous (i.v.)] and the selective neurokinin-1 neurotransmitter receptor antagonist aprepitant [125 mg, per os (p.o)] were administered 1.5 h before chemotherapy on day 1. Aprepitant (80 mg p.o.) and dexamethasone (8 mg p.o.) were administered on days 2 and 3. Prophylactic use of granulocyte colony-stimulating factor was not allowed.

The dose was modified for each patient based on hematologic or non-hematologic toxicity. Treatment was delayed if, on the planned day of treatment, laboratory results included the following: ANC < 1500/mm^3^, platelets <75,000/mm^3^, Hb < 9 g/dL, serum transaminase >100 U/L, total bilirubin >2.0 mg/dL, serum creatinine >1.50 mg/dL, or if symptomatic toxicity was present. In the event of National Cancer Institute Common Terminology for Adverse Events (NCI-CTC) grade 4 neutropenia/leucopenia, or grade 3 or higher febrile neutropenia/thrombocytopenia/diarrhea/stomatitis, the capecitabine and cisplatin doses were reduced by 1 dose level starting at the next cycle. Capecitabine and cisplatin doses could be reduced by 400 mg/m^2^/day and 10 mg/m^2^, respectively, for each level. Patients who could not tolerate cisplatin could continue to receive capecitabine monotherapy until disease progression or intolerable toxicity. Capecitabine could be reduced by 2 dose levels, but treatment was discontinued if subsequent reduction was indicated. In the event of grade 4 non-hematologic toxicities, treatment was definitively interrupted.

### Study assessment

Pretreatment evaluation included: medical history; physical examination; complete blood cell count and serum chemistry tests; esophagogastroduodenoscopy; and chest, abdominal, and pelvic computed tomography (CT) scans. Complete blood cell counts with differential and serum biochemistry analyses were repeated at each treatment cycle. Response was assessed radiologically every 2 cycles or when progression was suspected. The same radiologic method used to document disease at baseline was used at subsequent assessments. No independent radiologic review was performed. All adverse events experienced during the study were recorded and graded according to the NCI-CTC guidelines (CTCAE version 4.0).

### Endpoints and statistical analysis

The primary endpoint was overall response rate (ORR) according to the Response Evaluation Criteria in Solid Tumors (RECIST) criteria v1.0. Complete response (CR) and partial response (PR) were confirmed by reassessment on CT scans after at least another 4 weeks. Secondary endpoints included PFS, overall survival (OS), time to treatment failure (TTF), time to failure of strategy (TFS), and safety.

The previous phase III study showed that the ORR of XP for gastric cancer was 46% [8]. Furthermore, in the AVAGAST study, the ORR in the Japanese XP group was 49.2% [10]. Therefore, we calculated that 35 patients were required to achieve 80% power to reject the null hypothesis of ORR ≤ 30%, assuming that the true ORR was 50%, using a one-sided *α* of 0.05 based on the normal approximation for binomial distribution. Taking into consideration the dropout rate, the number of patients enrolled was 40.

The survival curve was estimated using the Kaplan–Meier method, and 95% CI was estimated using the Brookmeyer and Crowley method. Safety and efficacy analyses were both conducted on a full analysis set (FAS) population, which was defined as all patients enrolled in the study that fulfilled the eligibility criteria and received chemotherapy at least once. PFS was defined as the time from the date of enrollment to the first documentation of disease progression or death. OS was determined from the date of enrollment to the date of death or last confirmed date of survival. TTF was defined as the time from the date of enrollment to the discontinuation of protocol treatment, first documentation of disease progression, or death. TFS was defined as the time from the date of enrollment to second-line chemotherapy initiation, first documentation of disease progression, or death. All statistical analyses were performed with SAS version 9.4 (SAS Institute, Cary, NC).

This trial was registered with University Hospital Medical Information Network (No. UMIN:000006668).

## Results

### Patient characteristics

Forty-two patients were enrolled in this study from November 2011 to October 2013. Among them, 1 patient was excluded from all analyses due to failure to fulfill the eligibility criteria. Accordingly, 41 patients were included in the FAS population and analyzed (Table 1). Fifteen patients (36.3%) had undergone resection of the primary tumor: total gastrectomy in 9 patients and other surgeries in 6. Five patients had received prior neoadjuvant and/or adjuvant chemotherapy, while 36 patients had received no prior chemotherapy.

**Table 1:** Patient characteristics (*n* = 41)

Variable	*n*	(%)
Age (years)		
Median (range)	64 (50–81)	
Sex		
Male	34	82.9
Female	7	17.1
ECOG PS		
0	33	80.5
1	8	19.5
Primary tumor location		
U	12	29.3
M	19	46.3
L	10	24.4
Histology of primary tumor		
Well	18	43.9
Poorly	22	53.7
Other	1	2.4
HER2 status		
IHC 0/1+	37	90.2
IHC 2+/FISH negative	4	9.8
Disease status		
Advanced	28	68.3
Recurrent	13	31.7
Sites of metastasis		
Liver	17	41.5
Lungs	1	2.4
Lymph node	32	78.0
Peritoneum	12	29.3
Other	7	17.0

*ECOG* Eastern Cooperative Oncology Group, *PS* performance status, *HER2* human epidermal growth factor receptor type 2, *IHC* immunohistochemistry, *FISH* fluorescence in situ hybridization

### Treatment

At the data cutoff date, treatment was ongoing in just 2 patients. The major reasons for discontinuation of treatment in the remaining 39 patients were disease progression in 21 (54%) patients, adverse events in 11 (28%), surgical resection for the primary lesion or radiotherapy in 4 (10%), and other reasons in 3 (8%). Adverse events that required treatment discontinuation included digestive symptoms (anorexia/nausea/vomiting; *n* = 4), fatigue (*n* = 2), depressed level of consciousness (*n* = 1), hand–foot syndrome (HFS; *n* = 1), severe neutropenia (*n* = 1), renal impairment (*n* = 1), and a thromboembolic event (*n* = 1). The median relative dose intensity (RDI) was 83.7% [95% confidence interval (CI) 50.6–101.0%] for cisplatin and 70.1% (95% CI 48.6–109.2%) for capecitabine.

### Efficacy

**Fig. 1:**
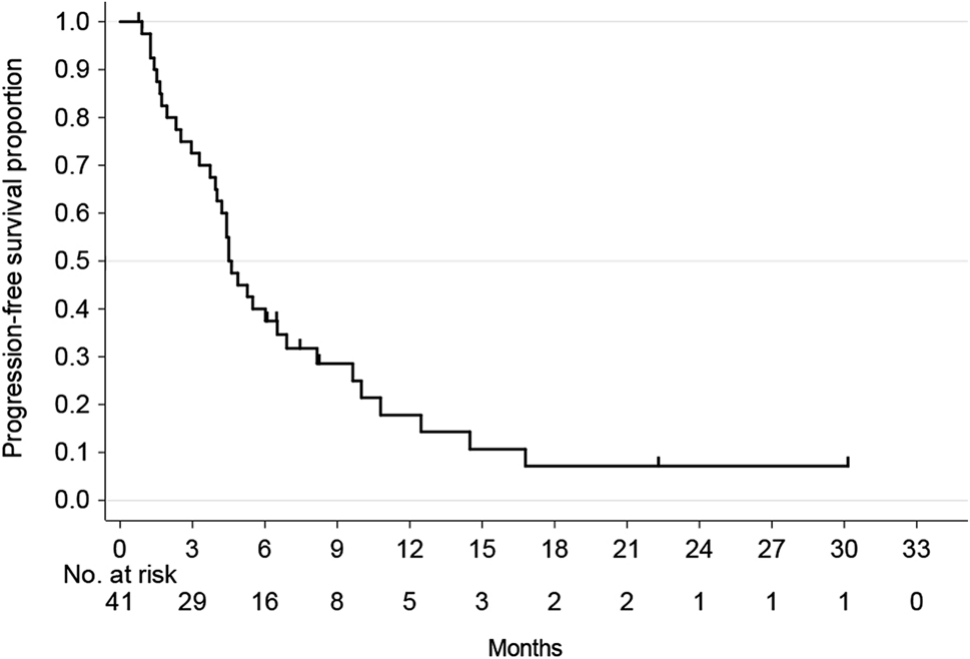
Progression-free survival (*n* = 41)

**Fig. 2:**
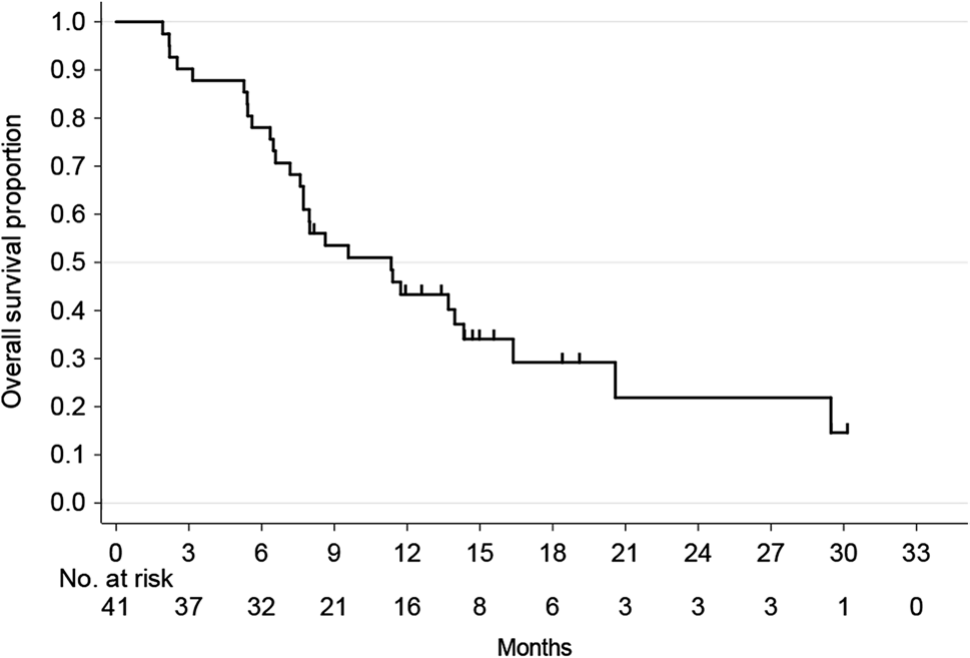
Overall survival (*n* = 41)

The ORR was 43.9% (95% CI 28.7–59.1%), with CR in 2 patients and PR in 16 patients (Table 2). The disease control rate was 78.0% (95% CI 65.4–90.7%). With a median follow-up period of 9.6 months (range 1.9–30.2 months), median PFS, median TFS, median TTF, and median OS were 4.6 months (95% CI 3.9–6.5), 4.4 months (95% CI 2.5–5.3), 4.0 months (95% CI 2.3–4.9) and 11.3 months (95% CI 7.7–14.3), respectively (Figs. 1, 2).

**Table 2:** Treatment response rate (*n* = 41)

Variable	*n*	% (95% CI)
Complete response	2	4.9 (0.1–16.5)
Partial response	16	39.0 (24.2–55.5)
Stable disease	14	34.1 (20.1–50.6)
Progressive disease	8	19.5 (8.8–34.9)
Not evaluated	1	2.4 (0.0–12.9)
Overall response rate	18	43.9 (28.7–59.1)
Disease control rate	32	78.9 (65.4–90.7)

*CI* confidence interval

### Safety

The worst toxicities experienced throughout the treatment period are listed in Table 3. The most common grade 3 or higher hematologic toxicities were neutropenia (37.5%) and anemia (24.4%). The most common grade 3 or higher non-hematologic toxicities were anorexia (24.4%) and nausea (12.2%). HFS occurred in 46.3% of patients; grade 3 or higher HFS occurred in 7.3% of patients. Febrile neutropenia occurred in 2 patients, but they recovered with conservative therapy and the protocol treatment could be continued. There were no treatment-related deaths.

**Table 3:** Maximum toxicity per patient (*n* = 41)

Adverse event	NCI-CTC grade					
	1	2	3	4	All (%)	3–4 (%)
Hematologic, *n*						
Leukopenia	8	15	7	0	73.2	17.1
Neutropenia	8	7	13	2	75.0	37.5
Anemia	13	17	10	0	97.6	24.4
Thrombocytopenia	20	3	1	0	58.5	2.4
Non-hematologic, *n*						
Alopecia	3	0	–	–	7.3	0
Anorexia	13	8	10	0	75.6	24.4
Depressed level of consciousness	0	0	1	0	2.4	2.4
Diarrhea	4	2	0	0	14.6	0
Dysgeusia	6	4	–	–	24.4	0
Fatigue	10	14	4	0	68.3	9.8
Febrile neutropenia	–	–	2	0	4.9	4.9
Hand–foot syndrome	10	6	3	0	46.3	7.3
Nausea	10	10	5	0	61.0	12.2
Peripheral sensory neuropathy	6	3	0	0	22.0	0
Renal impairment	7	0	0	0	17.1	0
Skin hyperpigmentation	13	0	–	–	31.7	0
Stomatitis	10	3	1	0	34.1	2.4
Thromboembolic event	0	0	1	0	2.4	2.4
Vomiting	5	2	3	0	24.4	7.3

*NCI-CTC* National Cancer Institute Common Toxicity Criteria

## Discussion

In this study, we found that the mXP regimen was active and tolerable as first-line chemotherapy in patients with mGC. Previous studies of the XP regimen in patients with mGC reported an ORR of 35–37.4% with a median PFS of 5.3–5.5 months and a median OS of 10.1–11.1 months [9, 12]. The efficacy in our study was equivalent to that of these previous studies, with an ORR of 43.9%, median PFS of 4.6 months, and median OS of 11.3 months, although the actual ORR was lower than the expected ORR (50%). To our knowledge, this is the first multicenter prospective study of capecitabine plus cisplatin as first-line chemotherapy for Japanese patients with mGC.

One reason for the lower than expected efficacy may be the comparatively lower dose intensity of capecitabine and the lower dose of cisplatin. In the AVAGAST study, the RDI of capecitabine was 80% in the Japanese XP group and 87% in the overall XP group. In contrast, with the mXP regimen, the RDI of capecitabine was 70.1%. As a result, the frequency of HFS in this study was lower than that in a previous report (57 vs. 46%) [10]. Lotions are currently recommended to improve moisturization and patients receive instructions on how to prevent adverse effects, such as heat exposure, and to reduce friction; therefore, the dose intensity is expected to be higher. This study shows that the mXP regimen maintained an RDI of cisplatin of 83.7 versus 71% in both the Japanese XP group and overall XP group in the AVAGAST study. This means that 16.7 mg/m^2^/week of cisplatin was actually administered in the mXP regimen, while 18.9 mg/m^2^/week was administered in the AVAGAST trial. Furthermore, treatment discontinuation due to adverse events occurred in only 6–7% in the Japanese XP group in the AVAGAST study, versus 28% in the present study. We planned the mXP regimen by modifying the cisplatin dose to improve dose intensity and ensure safety in the cohort of Japanese patients. However, the lower dose intensity of capecitabine, the lower dose of cisplatin, and treatment discontinuation due to adverse events may explain why this study did not reach the expected ORR. Table 4 shows a historical comparison of first-line chemotherapies consisting of capecitabine plus cisplatin for mGC.

**Table 4:** Historical comparison of first-line chemotherapy consisting of capecitabine plus cisplatin for metastatic gastric cancer

	Current study (*n* = 41)	AVAGAST, Japanese XP group (*n* = 94)	AVAGAST, overall XP group (*n* = 387)
Median OS (months) (95% CI)	11.3 (7.7–14.3)	14.2 (10.9–18.8)	10.1 (9.0–11.3)
Median PFS (months) (95% CI)	4.6 (3.9–6.5)	5.7 (5.3–7.0)	5.3 (4.4–5.6)
Response rate (%)	43.9	49.2	37.4
RDI of cisplatin (%)	83.7	71	71
RDI of capecitabine (%)	70.1	80	87.0

*AVAGAST* Avastin in Gastric Cancer, *OS* overall survival, *PFS* progression-free survival, *RDI* relative dose intensity

Compared with previous reports, severe toxicities were not commonly observed with the mXP regimen in the current study. In the AVAGAST study, grade 3 or higher adverse events in a Japanese XP group included neutropenia (48%), anorexia (29%), nausea (19%), and anemia (11%) [10]. In the present study, lower incidence rates of these adverse advents were observed: neutropenia, 37.5%; anorexia, 24.4%; nausea, 12.2%; and anemia, 2.4%. Moreover, about 50% of the Japanese patients in the AVAGAST study required a cisplatin dose reduction during the second treatment cycle, and 79.8% of patients required a cisplatin dose reduction due to adverse events at some point during the treatment course [10]. In contrast, grade 3 or higher toxicities during the first treatment cycle in the present study were neutropenia (*n* = 10), anemia (*n* = 2), thrombocytopenia (*n* = 1), anorexia (*n* = 4), nausea/vomiting (*n* = 3), stomatitis (*n* = 1), fatigue (*n* = 1), and febrile neutropenia (*n* = 2), while only 15 patients (37%) required a dose reduction during the second treatment cycle. In other words, the safety profile of the mXP regimen appears promising. On the contrary, mild bone suppression might mean that the dose of cisplatin is insufficient.

Several limitations of our study warrant mention. We did not perform a phase I study to determine the dose of cisplatin in the XP regimen for Japanese patients with gastric cancer, so the optimum cisplatin dose remains unclear. We set the cisplatin dose in the current study at 60 mg/m^2^ based on the S-1 plus cisplatin (SP) regimen [3, 13] and a previous phase 2 study of XP for gastric cancer [7]. The optimal dose of capecitabine, when administered as a monotherapy, is 1250 mg/m^2^ twice daily, on days 1–14, every 3 weeks [14]. Additionally, a previous phase 2 study administered capecitabine at 1250 mg/m^2^, twice daily, on days 1–14 plus cisplatin at 60 mg/m^2^ i.v. on day 1, repeated every 3 weeks [7]. However, the standard XP regimen for mGC now consists of capecitabine (1000 mg/m^2^, twice daily, on days 1–14) plus cisplatin (80 mg/m^2^ on day 1) every 3 weeks [8]. This means that the standard XP regimen consists of a decreased capecitabine dose and an increased cisplatin dose, compared with previous studies. When combined with cisplatin, both S-1 and capecitabine are orally administered with fluoropyrimidines to ensure efficacy in the treatment of gastric cancer. However, the recommended dose of cisplatin in a combination treatment depends on the combination: 60 mg/m^2^ cisplatin for the SP regimen and 80 mg/m^2^ cisplatin for the XP regimen. Furthermore, the present study did not reach its primary objective. This might mean that the anti-tumor effect of cisplatin in the XP regimen is more dose-dependent than that of cisplatin in the SP regimen, so we should have discussed the modification of cisplatin dose further. Preclinical studies demonstrated the indirect role of cisplatin as a modulator for fluorouracil (5-FU), in addition to its direct effect as an effector [15, 16]. S-1 is a novel oral fluoropyrimidine consisting of a 5-FU prodrug, tegafur, a dihydropyrimidine dehydrogenase inhibitor, 5-chloro-2,4-dihydroxypyridine, and the orotate phosphoribosyl-transferase inhibitor, potassium oxonate, which suppresses the gastrointestinal toxicity of tegafur [17]. In contrast, capecitabine is an oral fluoropyrimidine that is metabolized primarily in the liver and converted in tumor tissues to 5-FU by the enzyme thymidine phosphorylase, which is present in higher concentrations in tumor cells than in normal cells [18]. The differences between S-1 and capecitabine may affect the role of cisplatin in each regimen. In the present study, only 1 patient did not experience grade 3 or higher toxicities during treatment; in other words, this patient might have been disadvantaged by the mXP regimen because the cisplatin reduction may have been unnecessary.

In conclusion, although the present study did not reach its primary objective, the mXP regiment showed promising efficacy and an acceptable tolerability profile in clinical practice. The modified XP regimen is a treatment option for patients with mGC.
